# A Clinical Competency Framework for the Basic Package of Oral Care: Perceptions of Primary Oral Health Providers in Rural Nepal

**DOI:** 10.3389/fpubh.2022.914581

**Published:** 2022-07-14

**Authors:** Bidhya Koirala, Shreedhar Acharya, Laura Spero, Rakhi Mittal, Daniel J. Erchick

**Affiliations:** ^1^Jevaia Nepal, Pokhara, Nepal; ^2^Independent Research, Sydney, NSW, Australia; ^3^Department of International Health, Johns Hopkins Bloomberg School of Public Health, Baltimore, MD, United States

**Keywords:** competency framework, Basic Package of Oral Care, quality of care, Nepal, dental education, Primary Oral Health Provider, quality assurance

## Abstract

**Introduction:**

The basic package of Oral Care (BPOC) was developed to improve oral health care for underserved populations worldwide. However, systematic delivery of the BPOC has been difficult to achieve, and training efforts have in some cases contributed to proliferation of malpractice. Standard Competency Frameworks (CF), increasingly used in dental and medical education to improve quality assurance, have not been established to date for the BPOC.

**Methods:**

To evaluate provider perceptions of a BPOC-specific CF, in-depth interviews were conducted with 7 Primary Oral Health Providers (POHPs) and 5 Clinic Assistants working in the Jevaia Oral Health Care project (Jevaia) in Nepal. Participants were limited to providers who have used the CF. Interviews were audio recorded, transcribed in Nepali, and translated into English. A qualitative thematic analysis was applied through a multi-stage review process, and emergent themes were further grouped and categorized to draw final conclusions.

**Results:**

Findings were categorized into four groups: (1) “What is the CF to Me”: Respondents frequently conflated the CF with professional development training. These activities together were essentially felt to offer clear performance guidance and a pathway for learning. (2) “Relationship to the Work”: Respondents reported that the CF's guidelines increased confidence, peer accountability, and job satisfaction. (3) “Practical Improvements”: Providers felt the CF improved their clinical skills, communication, crowd management, and teamwork. (4) “Community Impact”: Many participants felt that improved skills had led to a more efficient workflow, greater community acceptance, and increased utilization of services.

**Conclusions:**

Clinicians broadly felt that the CF improved both their professional satisfaction and the quality of patient care. CFs should be considered integral to BPOC implementation, along with opportunities for continuous professional learning, and these activities will likely be most meaningful and impactful when recognized by government and other licensing bodies.

## Introduction

Oral diseases are among the most common non–communicable diseases worldwide, with a higher burden occurring in low- and middle-income countries (LMICs) (1). Oral health care has been recognized as an essential component of universal health coverage (2). However, global access to oral health care is insufficient, particularly at the primary care level in remote areas and LMICs. Recognizing this critical gap, in 2021 the World Health Organization adopted a resolution calling for the creation of a global oral health strategy by 2022 and an action plan by 2023 (3, 4).

Lack of access to primary-level oral health care, especially in remote and impoverished regions of the world, has long plagued efforts to reduce the global burden of oral disease. To address this problem, Basic Package of Oral Care (BPOC) was developed in 2002 by the World Health Organization Collaborating Center in the Netherlands (5). The BPOC is a package of clinical techniques designed to be carried out by a flexible health workforce, including midlevel providers without a prior dental specialty. It includes (1) Oral urgent treatment (OUT), simple tooth extraction with local anesthetic used for pain relief and emergency management (2) accessible fluoride toothpaste, and (3) atraumatic restorative technique (ART), a method of filling cavities with hand-mixed glass ionomer cement. Since the original development of the BPOC, it has sometimes been modified with contemporary additions such fluoride varnish or silver diamine fluoride (SDF), a topical silver-fluoride mix that can be used to non-invasively arrest and harden carious lesions (6).

The BPOC has been noted for its potential to be strategically combined with mainstream dental care in national health systems seeking to achieve Universal Health Coverage (UHC) (7). Yet since 2002, there have been few examples of routine BPOC delivery by midlevel providers in remote health care facilities worldwide. Implementation strategies have largely focused on single-instance training of midlevel providers working at the community level. But there is little translational evidence showing this approach has led to sustained, effective BPOC services or integration with national healthcare systems.

One barrier to systematic and stable implementation of the BPOC is the absence of standard competency frameworks and processes for maintaining quality assurance. Competency frameworks are increasingly used in medical and dental education, as test-based training has shown a tendency to promote rote learning that often does translate to high-quality practice (8–10). Competency testing refocuses learning away from memorization of facts and toward critical thinking and synthesis of knowledge in real clinical situations. Khanna et al. (8) suggest that “competency includes knowledge, experience, critical thinking, problem-solving skills, professionalism, ethical values, technical and procedural skills”.

While a small number of oral health-focused competency frameworks have been proposed for midlevel health professionals, (9) these are not necessarily BPOC-specific and tend to encompass a sweeping range of skills that are difficult to measure or standardize. In 2015, a multidisciplinary group developed a preliminary Oral Health Competency Framework that recognizes the important role of the BPOC and includes 23 competencies for community health professionals (11). A 2016 guideline made for African countries outlines “minimum competencies” for the BPOC, but these are hard to quantify, proposing standards such as “Maintain dental equipment and materials” (12). Other competency frameworks are even more far-reaching, offering guidelines for Universal Health Coverage generally (13). Such frameworks offer no measurable competency assessments for BPOC clinical practice or for essential clinical skills such as differential diagnosis and treatment planning. Although broad frameworks are useful in thinking about a comprehensive approach to reduction of oral diseases, they leave BPOC providers without rigorous clinical standards to provide guidance and accountability at the point of care.

Nepal was one of the first LMICs to offer BPOC training for midlevel providers in rural government health facilities, beginning in the early 2000s, in collaboration with the United Mission to Nepal's Oral Health Program (14). Since then, BPOC training for midlevel providers in Nepal has varied greatly in content and format, reflecting a lack of standardization for BPOC training worldwide. After over 20 years of ad-hoc BPOC instruction for community health professionals in Nepal, there is no systematic delivery of the BPOC in Nepal's rural Health Posts, and no health policy specifying the role of the BPOC in the health care system. In the same time period, dental caries has become a widespread public health concern for adults (15) and one of the most prevalent non–communicable diseases among Nepali children (16, 17).

On the other hand, BPOC training in Nepal *has* led to a proliferation of malpractice. Unregulated healthcare professionals with BPOC training, working in high-need areas without adequate supplies or supervision, have deployed their skills almost exclusively in private clinics, and often expanded their scope of care beyond the BPOC to unlicensed dental practice. This trend has sparked objections from mainstream dental professionals in Nepal, who, pointing to safety and quality of care concerns, have vigorously advocated to shut down BPOC training in Nepal entirely (18). The BPOC has met a similar fate in many regions of the world where it has been tried, with haphazard implementation and unreliable quality assurance eliciting opposition from conventional dental professionals (19–22).

The absence of accepted competency frameworks thus represents a vital gap in achieving the vision of the BPOC as a building block of universal oral health coverage. Jevaia Oral Health Care (Jevaia), a Nepal-based non-profit, has implemented a BPOC Competency Framework (CF) in community-based oral health programs in Kaski, Parbat, and Lamjung districts since 2018. This investigation aims to evaluate how Primary Oral Health Providers (POHPs) delivering the BPOC in Jevaia's programs felt that the CF impacted their work. The results presented should inform efforts to expand access to primary-level oral health services by deploying the BPOC in remote and underserved areas around the world.

## Methods

This was a qualitative study, based on in-depth interviews, of how POHPs and Clinic Assistants working in the Jevaia Oral Health Care Project viewed a BPOC Competency Framework (CF) that had been used for 2.5 years. Jevaia operates in coordination with government bodies in Kaski, Lamjung and Parbat Districts of Nepal to provide technical support to BPOC operators, and is registered with Nepal's Social Welfare Council (#42008). This study received ethical approval from the Nepal Health Research Council (Kathmandu, Nepal) (Reg #324/2021P, approval ref #282) and an exemption as non-human subjects research from the Institutional Review Board at the Johns Hopkins Bloomberg School of Public Health (Baltimore, United States).

The CF was developed in 2018 by an experienced dental public health clinician (Dr. Bathsheba Turton, BDS, MComDent, PHD). The CF consists of a series of checklists related to clinical technique. It is used together with the following annual practicing requirements for certification renewal: professional development hours, documentation of peer contact, practicing hours, and viva testing. These activities incorporate applied clinical reasoning and non-clinical areas of practice (see Supplemental Material section 1). At the time of this study, the CF had been implemented in Nepal by Jevaia for 2.5 years. Jevaia also conducted professional development training for all clinicians, including Assistants, every 6 months from 2017–2021, reviewing topics and clinical skills within the CF, as well as a broad range of skills not explicitly covered by the CF but applicable within the BPOC scope of practice.

### Participants

Participants included POHPs and Clinic Assistants who are jointly employed by Jevaia Oral Health Care and local government bodies, with technical support provided by Jevaia. POHPs have formal education as Community Medical Assistants (CMA), an 18 month credentialing course, followed by supplemental training in the BPOC. Clinic Assistants are support staff who may or may not have previously come from a health background and are responsible for supporting POHPs during provision of clinical services. In the normal course of their work, POHPs and Assistants deliver the BPOC for patients of all ages in government Health Post clinics, schools, and community spaces.

All study participants began working with the BPOC-CF in August 2018. Prior to this, all POHPs received certification in the BPOC by outside agencies. Four POHPs were trained in the BPOC anywhere from 3–10 years prior to implementation of the Competency Framework. One POHP was exposed to the CF during their initial BPOC training in 2018.

### Data Collection

To evaluate clinicians' perceptions of the impact of the CF on their work, in-depth interviews lasting 60–90 min were conducted with 5 POHPs and 7 Clinic Assistants, between August 2021 and September 2021. Investigators aimed to include all Jevaia clinical staff as participants in this study. No other similar government health agencies or NGOs have adopted the same Jevaia's CF used here; hence, sample size was limited to this study population. Interviews were conducted in-person in the areas where Jevaia services are provided, specifically Kaski, Parbat, and Lamjung districts.

The principal investigator (PI) (BK) and co-investigator (Co-I) (SA) conducted the interviews with participants in-person using different discussion guides for POHPs and Clinic Assistants (see Supplemental Material section 3). Written consent was taken by the interviewer before the start of the interview. Interviewers stressed to participants that they were not required to participate and that neither their participation nor their responses would impact any aspect of their employment. Interviewers audio recorded the discussion. The discussion guide included questions in five sections: (1) roles and responsibilities (2) use of the CF (3) CF relationship to professional experience, community acceptance, and clinical care (4) challenges faced by the participants, and (5) recommendations.

### Analytical Approach

**Figure 1:**
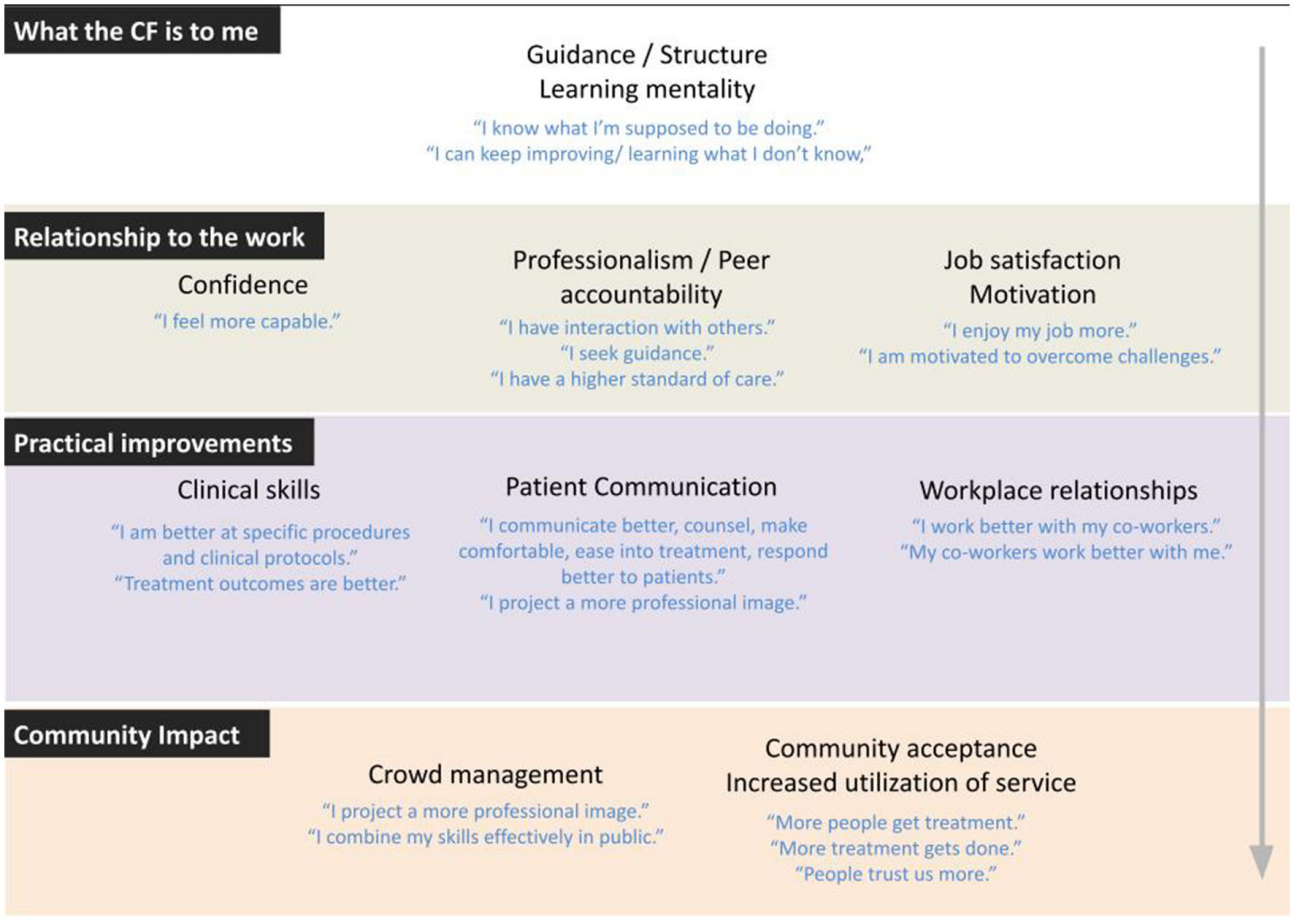
Thematic Groupings of Provider Perceptions of BPOC-CF.

A professional translator transcribed audio files in Nepali and translated them into English. The PI and Co-I checked sections of each transcript for accuracy and consistency of the data. Investigators applied a qualitative thematic analysis and multi-stage review process to analyze the data. First, an a priori code structure was developed based upon existing literature and prior observation of routine program activities. Then all team members individually reviewed and coded a single transcript. All authors met to discuss their results and refine the code structure. The team subsequently coded a second transcript using the new code structure. In the second discussion, the team finalized the code structure and meanings of the codes (see Supplemental Material section 2). Then the PI and Co-I applied the final code structure to all of the transcripts. In the last stage, themes emerging from the data were organized once more into a series of new groupings (Figure 1). Figure 1 was finalized with the full team and applied to draw final conclusions.

## Results

Overall, respondents described several ways in which they felt the CF improved their skills, professional collaboration, and job satisfaction. Following the analysis of all interviews using the a priori code structure, the original 10 themes were reorganized as sub-themes of four larger concepts. Some original themes were further divided into new categories to better demonstrate a diversity of ideas that emerged within them. Figure 1 was developed to present results with the revised thematic structure, and contains four groupings: (1) What the Competency Framework is to Me, (2) Relationship to the Work, (3) Practical Improvements, and (4) Community Impact.

### What the Competency Framework Is to Me

“*We move our feet even on roads we have never taken.”* 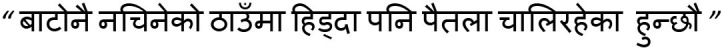

Almost all respondents made no distinction between “competency framework” and “professional development training.” Rather, their experience with the CF was largely intertwined with the training experience, whether in regard to the competencies explicitly included in the CF or a variety of other clinical skills related to core competencies in the CF. Some Assistants could not recall what the CF was, even though all had participated in competency-related training using the CF alongside POHPs. Most respondents referred in their interviews to “training” even when talking about the CF, and some effects that respondents attributed to the CF may have been more accurately associated with experiences and adjacent content in bi-annual professional development workshops. Results, therefore, tend to reflect perceptions of both activities as interchangeable experiences.

The CF and professional development training were perceived to serve two core functions (Table 1): establishing clear standards and fostering a learning mentality. Combined with a training schedule, the CF was perceived mainly as a source of structure and guidance. Well-defined performance targets led clinicians to feel that the process of gaining competency over time had become more structured and achievable. To some degree the CF elicited a sense of “I know what I am doing,” but even more so “I know what I am supposed to be doing” and “I know how to figure out what I don't know.”

**Table 1:** What the Competency Framework is to me.

Clarity of standards	•*What I feel about this framework is that while walking on a black, paved, straight road it might seem like we don't get very far. But if we walk kilometer- by- kilometer then we will reach the destination as planned without any wastage of time. (POHP)* •*After the CF training, I started doing things step- by- step. (POHP)*
Learning mindset	•*What I think is, whenever we work, we work in the same way that we are used to working. This competency training will show us how we can work by different ways or mediums or methods. (POHP)* •*I used to do it in my own way but later after the training, I knew about the ways of asking the question and carrying out treatment accordingly. (POHP)*

### Relationship to the Work

“*Words used to disappear from my mouth.”* 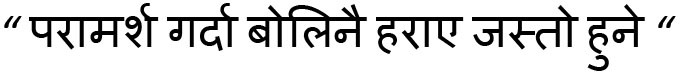

The establishment of a set of guidelines had multiple effects on participants' relationship to their work. These included more confidence, more peer accountability, and more job satisfaction and motivation (Table 2).

**Table 2:** Relationship to the work.

Confidence	•*It was really difficult at the start as I had never done this work. One week of training was not enough. My hands used to tremble, and I couldn't even look at the patient's face as I used to be shy. Words used to disappear from my mouth. If it was another type of disease, I could have worked from afar. Dental service requires close contact. As I started participating in various training, I felt comfortable gradually to speak, to treat even the male patients.” (POHP)*
Peer accountability	•*In a situation when I didn't know something or was confused or not sure of something, it helped me to ask my seniors or friends. I could effectively talk with them. (POHP)*
Job satisfaction & motivation	*Intellectual stimulation* •*If you receive training and utilize it for a long time it becomes an old technique. For example, if you start something in the morning it will be so fresh and energetic, slowly in the evening one's level of energy goes down. Similarly, if I received training 12 years back and continue working the same way I won't learn new things and I might forget many things also. We need refresher training to update our knowledge and motivate us to work. (POHP)* *Collegial support* •*We will have a feeling that “I can do” when we see our friends doing things we find difficult. It motivates us to do such difficult work, which allows us to try and learn new things. (CA)* •*We met friends from different places and shared our experiences. They knew the things which we didn't know, and we knew the things that they didn't know. (POHP)* *Competence-related fulfillment* •*I used to be scared the POHP would notice faults in my work. Now after the training, I know where and how to keep things properly. I also know how to choose the right tool for the right treatment. I felt this made my work easier and comfortable. (CA)*

#### Confidence

“*My Hands Used to Tremble.”* 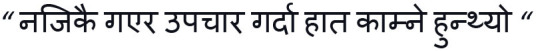

Clinicians expressed new confidence in their ability to perform specific techniques, such as ART and extraction, and also demonstrated more confidence in their interpersonal interactions with patients and with each other. Specifically, POHPs felt more confident in their ability to work efficiently, to put patients at ease and provide oral health counseling, and to manage crowds in community settings. There was also increased confidence around working in isolation and managing risk of adverse events. A female POHP noted newfound confidence in working at close distance with male patients. Among Assistants, many respondents reported an improved understanding of the clinical workflow that had made them more confident around the POHP and more willing to speak around patients.

#### Peer Accountability

“*We discuss among friends.”* 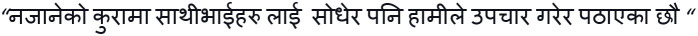

Participants shared a variety of ways in which their professional interactions increased, leading to more guidance-seeking and the internalizing of a higher standard of care. Clinicians reported that the CF strengthened their connection to a professional community, especially when paired with professional development training. Peer-to-peer contact was felt to foster professional dialogue and group problem-solving. Some respondents recounted specific cases where they had delayed treatment to ask peers for second opinions.

Although Assistants had not been directly tested on the CF, many Assistants also reported that professional collaboration during training had enhanced their clinical knowledge, standard of care, and accountability to continual learning.

#### Job Satisfaction and Motivation

“*If I learn new things, it will motivate me.”* 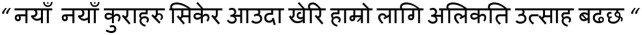

Respondents reported that being more confident with patients and more connected with peers had enhanced morale and raised job satisfaction. These effects were related to intellectual stimulation, collegial support, and competence-related fulfillment, or a sense of enjoyment around being capable. POHPs reported feeling more motivated to perform their responsibilities effectively, and Assistants widely stated that better clinical knowledge and more confidence working with the POHP made their work not only more effective but also less stressful and more enjoyable. Additionally, respondents felt that the opportunity to learn and to pursue a standard of care was itself a source of refreshment and motivation. The CF was thus seen to represent not only a set of requirements, but an opportunity for personal growth that led to a more fulfilling professional experience and professional identity.

### Practical Improvements

“*There is a difference between knowing and doing things practically.”* 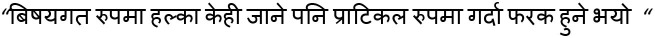

Clinicians raised a variety of ways they felt competency testing and bi-annual training had made for practical improvements at the point of care. These can be subdivided into clinical skills, communication, and workplace relationships (Table 3).

**Table 3:** Practical improvements.

Clinical skills	*Direct treatment skills* •*It's not easy to work practically without proper training. There are things like how to dry up the teeth or apply cotton or how to extract the teeth, hold forceps, how to ensure sterilization. There is a difference in knowing and doing things practically. (POHP)* *Infection control* •*We used to clean the clinic, instruments, chairs and other things that we use before the training, but we didn't do it regularly. After the training, we are doing all these activities regularly as a mandatory thing. (CA)* *Differential diagnosis & referral* •*There has been a huge improvement in the quality. We didn't know when to do the filling, which has improved. As in, in this case, this should be done. We do things that we can and refer cases we can't deal with. (POHP)* •*Before the training, the POHP I work with used to try to treat all sorts of cases though he didn't know how to. He was not in favor of referring to the hospital and tried to do it himself. After the training, a lot of changes have been seen. He refers many patients to the hospital and encourages them to go. (CA)* *Approach to care* •*We used to go there with a fixed responsibility just to extract the teeth, fill the teeth. After this training when we started to work for certification I started to see the patient from a different perspective. Patients are God. (POHP)* •*After going into the competency framework there has been a perspective change of us toward patients. (POHP)*
Patient communication	•*When a child sees a man with a white coat, the child gets scared. We have to build a capacity to deal with such cases. (POHP)* •*We clear all their doubts and ask them how they are feeling. We make them really confident and drive their fear away. (CA)* •*The POHP also kindly talks with the patients. He asks everything in detail. He asks them how they are feeling? How long have they been facing the problem? He behaves very well to the patients. (CA)*
Workplace relationships	•*I used to think that I should do my work alone. I used to think that we all have separate work, and we should do it separately. But after the training, we started doing it collectively even while visiting the community. It made our work easier and better. Likewise, there was no debate about the work. There was cooperation. (POHP)* •*Before we needed to show and teach which equipment needed to be brought to us. But now if I say to bring ART equipment, the assistant recognizes it and brings that equipment. (POHP)* •*After attending the 3 weeks of training, it was much easier to work. I also felt comfortable working with the POHP as I knew his working procedure, which was easy to follow. (CA)*

#### Clinical Skills

“*The training has brought changes in our working nature and procedure.”* 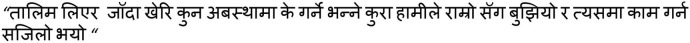

Respondents described many BPOC-related skills that they felt had been enhanced by the application of competency testing and training. These included infection control, direct treatment skills, differential diagnosis, and overall approach to care.

Perceived improvements to safety and infection control included surface disinfection, sterile/non-sterile zone integrity, general clinic organization, and use of safety glasses. In the treatment interaction, clinicians reported increased competence with history-taking, clinical documentation, differential diagnosis and referral, treatment planning, risk-management, pain management, and patient follow-up. Clinicians also felt they had increased the proportional use of non–invasive techniques (especially counseling and SDF) and reduced their use of extraction. They reported better ability to decide between use of Fluoride Varnish, SDF and ART, and better technique in executing these skills; for example, tooth preparation prior to GIC placement. One respondent reported observing an increase in survival of ART fillings. Other clinical skills mentioned by participants were combination use of ART and SDF, moisture control, administration of local anesthetic, and forceps handling. There was a feeling expressed that the approach to care had become more patient-centered and comprehensive.

Clinicians reported more competence with incorporating medical conditions common in the regions where they work, such as high blood pressure, asthma and thyroid problems, into assessments, treatment plans and patient counseling. Clinicians also felt better equipped to manage adverse events and emergencies, both due to direct training in emergency management and through access to a professional network of colleagues. Both POHPs and assistants referenced better adherence to scope of practice and increased referral to higher care.

#### Patient Communication

“*It has brought a lot of changes in my working style.”* 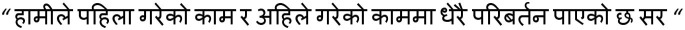

Communication was a major topic across all interviews and codes. All clinicians felt that after training and application of the CF, POHPs were better able to communicate with patients about diagnoses and more effective at alleviating fear and anxiety. Assistants observed these changes in their partner POHPs. Better communication was widely perceived by respondents to increase quality and safety of care. Participants reported taking more time to record patient history and risk factors, and more careful treatment-planning based on patient interviews and demographic characteristics.

#### Workplace Relationships

“*There is no debate about the work.”* 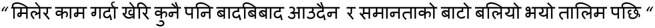

Workplace relationships between POHPs and Assistants were seen to have been markedly strengthened by professional training and the process of working toward clinical competencies as shared performance targets. Assistants, especially, both felt and were perceived by POHPs to be more comfortable and effective in their roles due to improved ability to identify instruments and a more rigorous understanding of clinical procedures, allowing for prompt and hygienic delivery of instruments in line with competency protocols. Clinicians also reported increased camaraderie and cooperation, especially when multiple clinical teams work together in a crowded setting.

#### Community Impact

“*Now they come searching for us.”* 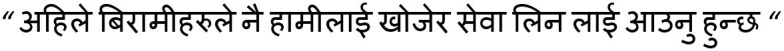

Providers felt that community acceptance of the oral health service had improved overall, and that utilization of services had increased (Table 4). Many participants felt that their improved communication skills, particularly explaining diagnoses and alleviating fears, had led to more willingness among patients to accept treatment once in the chair. Clinicians also felt that better proficiency with communication, treatment planning, and triaging cases among high volume of patients had made a more efficient workflow. This was especially relevant at crowded community programs, where clinicians perceived reduced frustration and confusion among patients, leading to more positive views of the oral health service generally. One Assistant shared the perception that “people have a different feeling about the service we provide” due a shift away from working “recklessly” and toward publicly visible improvements in infection control practices. Assistants and POHPs alike reported that the flow of patients had increased over time, though it was not always clear to what extent they felt this was related to implementation of the CF specifically.

**Table 4:** Community Impact.

Community acceptance	•*Now we'd done the program in a much more easy manner and everyone got checked easily. This is something I felt happy about*. •*There is a perspective change of society while working. (POHP)*
Increased participation	•*Initially we used to have very limited patients but now we receive all patients from this village and even many people come referred by other places. (CA)* •*The training made me comfortable that if the counseling is provided in this way, the patients are convinced to carry out the treatment. (POHP)*
Crowd management	•*After the training, I learned not to get panicked while working in the community in front of a large number of people. (POHP)* •*I knew after the training that it was not possible to do all the work right in the field. which made my work more comfortable and effective. The patients also understood it and it was advantageous for both. (POHP)* •*Patients used to enter all at once, making a crowd, and they used to complain. Now, we keep them all organized. They talk about these changes with each other. (CA)*

Some non-competency factors were raised in relation to community acceptance. Compared with city-based dental care, POHPs felt the community was accepting of their care for reasons such as consistency, the convenient availability of community-based care in schools and public spaces, proximity of services as compared with urban hospitals, and increasing visibility and familiarity with clinical personnel over time.

### Challenges

“*So should we take their history or treat them in ten minutes??”* 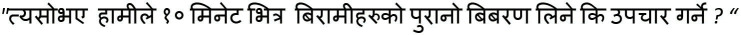

Respondents identified a variety of operational and credentialing challenges that they felt the CF either could not address or made harder. Operationally, in schools and other community settings with high patient volume, clinicians felt that sometimes time pressure made it difficult to implement knowledge gained through the CF. As one POHP put it, “Sometimes patients come in a hurry, saying ‘Someone is babysitting my child,' or breastfeeding a toddler and saying ‘I have to go home in 10 minutes.' So should we take their history or treat them in 10 minutes? We get confused.” It was also felt that communities often carry an expectation of dental extraction and that patients may feel disappointed by more gentle interventions even when indicated by competency-based protocols. Clinicians expressed concern about the accuracy of patient self-report when giving medical histories, as application of the CF requires obtaining a correct history to assess risk and make a treatment plan. The CF does not address public understanding of oral disease, and some providers worried that they could be blamed for poor care when patients continue to experience oral disease secondary to diet and other lifestyle drivers. “No matter how good our work is,” one POHP put it, “if the patient doesn't follow instructions, it won't work and then we must bear the blame.”

The other area of difficulty raised by clinicians was around official recognition of the Competency certification. Despite many benefits that respondents felt the CF did provide with regards to their perceived legitimacy, there remained the issue of inadequate formal credentialing. “People here think that as we have not done dental studies, we are just common POHPs. That is possibly the reason, I think, behind their doubt,” said one participant. For all its benefits, the CF was felt to have limits in helping providers overcome public perception of sub-standard formal licensing.

#### Recommendations

“*This organization has taught us many things, but hasn't given us any rights.” (POHP)* 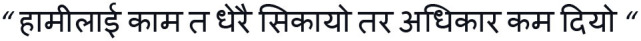

When asked for recommendations, many participants commented on general ideas around the oral health service, such as suggesting that the BPOC should be provided in Health Posts, that the public should be made more aware of the importance of oral health, that preventative care and reduction of lifestyle risks should be made common, and that consistent training should be made available to POHPs and Assistants.

A few participants, however, gave recommendations around certificate validity and job security. Respondents emphasized the need for government recognition of the Competency certification, and a need for official POHP (i.e., BPOC specialist) jobs in the government system. Presently, Jevaia's Competency certificate has no official validity outside of the Jevaia Nepal agency, and a POHP's professional experience is not officially recognized by any official body even after many years of practice. The absence of any official recognition of Competency maintenance was felt by some participants to undermine the usefulness of a Competency certification as it offers no meaningful employment security. “Even with a competency certificate,” stated one POHP, “the work is the same and there is no upgrade in the post. So there is no difference between taking and not taking the competency certification.”

Relatedly, participants felt that the functionality of a Competency certification was tied to the presence of BPOC-specific jobs in the healthcare system. “The experience I have and this certificate are not something you just store in a cupboard. If you could clarify where we can use the certificate then we would be grateful,” pointed out one respondent. Clinicians impressed that their competencies are only useful if the job market is seeking those competencies, and therefore “if an official post is added and there is a sanctioned position for the POHP, only then will the competency have a benefit.”

Similarly, participants recommended that professional development training—a broader quality assurance measure that overlaps with competency testing—be conducted through a nationally certified training agency. Like the CF, participants felt that continued professional development should carry the benefits of official standing. As one participant put it, “We just want to have a validation of our learning from the government.”

## Discussion

This was a qualitative study of in-depth interviews with clinical providers who have been using a CF for 2.5 years as part of delivering the BPOC for rural communities in Nepal. The CF was perceived by POHPs and Clinic Assistants to help them deliver better care, continuously address practice questions, collaborate with each other more effectively, and communicate better with patients. Notably, the CF itself was often conflated with professional development training, which has been delivered alongside competency testing and covered an array of practice-related topics beyond those directly addressed by the CF.

The absence of competency standards has likely contributed to significant variability in the delivery and quality of BPOC implementation worldwide. Since 2002, each agency, government or independent group that has attempted to deliver the BPOC has been left to develop its own training, quality of care guidelines, and assessment tools, if such assessment is done at all. Existing CFs for community level health professionals delivering the BPOC in low resource settings tend to cover a very broad range of non-technical, largely subjective competencies (11–13), raising questions about specificity, standardizability, and feasibility of implementation.

Perhaps one reason for this tendency toward the ideological is that CFs tend to be developed from high-level perspectives to meet sweeping public health goals. How CFs are perceived by community level health personnel at the point of care has not been well examined. This viewpoint is particularly important when it comes to frontline healthcare providers serving remote and low-resource settings worldwide, often in relative isolation and on the periphery of established health care systems and supports.

The CF evaluated in this study was comparatively focused, technical, and measurable: it consists of a series of checklists used to evaluate execution of BPOC clinical techniques. It is used with annual recertification requirements for professional development training, peer contact, practicing hours and viva testing. POHP perceptions of the CF may be broadly understood as falling under two umbrellas: directly skill-related/practical and more generally profession-related.

At a practical level, the CF was felt to strengthen understanding and execution of BPOC techniques and to have improved quality of care. Improvements were felt to be not only at the procedural level, but throughout the patient consultation. Providers even felt that these enhanced clinical abilities led to more positive community perceptions and more participation in the oral health service.

In addition to improved practical knowledge, findings revealed that the process of applying the CF influenced the way providers felt about their work. Stronger professional relationships, systematic methods of learning and troubleshooting, and better confidence, teamwork, and patient relations appeared to foster a more positive professional identity and increased job satisfaction. Many of these qualities have also been identified as core competencies for midlevel dental professionals (9). What is interesting is that these felt professional competencies were mostly achieved indirectly, through implementation processes associated with a narrower, clinically-focused CF.

**Figure 2:**
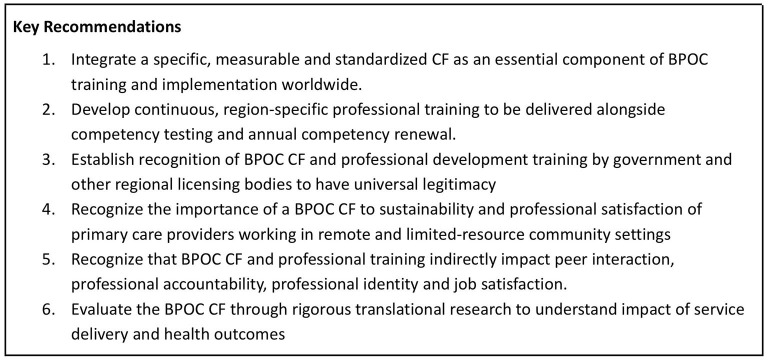
Key Recommendations.

Present findings suggest the need to rethink the core functions of a BPOC Competency Framework (Figure 2). Low job satisfaction and high levels of isolation and malpractice are serious problems in providing high quality rural health services (23–25) and these problems have especially plagued expanded use of the BPOC. It is therefore meaningful that the benefits experienced by clinicians from the CF went beyond both public health interests and purely technical knowledge, contributing to intangibles that are necessary for sustainability and quality of services, most notably around the rigor and resilience of the provider's role.

The CF might therefore be considered relevant at both individual and systemic levels. Health professionals specializing in the BPOC are likely to work remote and impoverished areas with variable needs and conditions, and CFs apply universally to all practitioners by embodying professional standards of care. CFs also reallocate some of the burden of care quality from individual providers to the profession as a whole. These findings suggest that a BPOC competency framework can function not only to hold oral health professionals to account, but to provide a supportive scaffolding within which providers can flourish through lifelong learning.

### Challenges With the CF

Respondents identified a variety of challenges that they felt the CF either could not address or made more difficult. Many were related to chronic situational factors such as time pressures, accuracy of patient self-report during assessments, patient compliance with lifestyle/diet recommendations, expectations created by previous service delivery patterns, and arduously transporting supplies by foot to field settings. Future improvements to the CF might seek to address some of these challenges, but many will also require systemic interventions that tackle root causes of poverty.

However, one challenge that stood out was the lack of formal recognition of the CF by a governing body. While participants did not have many recommendations around the internal structure of the CF, some felt strongly that its utility was critically undermined by its limited validity in the formal health care system.

These findings call attention to the fact that historically, BPOC training has often been delivered as a one-time add-on for community level health professionals with primary credentials obtained through parallel formal channels. This approach has arguably emerged as a serious flaw in the approach to systematic delivery of the BPOC by midlevel providers in community settings. The feedback from PHOPs in this study suggests that the importance of ongoing training and competency testing that is officially sanctioned (and even required) by official bodies is critical to the viability of the professionals delivering that care–and by extension, the efficacy and safety of BPOC services.

This study offers insight into a largely under-studied area around the development of competency frameworks and the dissemination of the BPOC: the perspective of frontline health professionals working in limited resource settings. Its strengths were in the uniqueness and authenticity of the vantage point that was voiced. A major limitation was the small number of participants and the risk of bias as primary investigators work in the same organization as respondents. This bias was managed by bringing in two investigators with no ties to Jevaia Oral Health Care, by specifically asking respondents for criticisms and suggestions regarding the CF, by de-identifying data and ensuring anonymity, and by applying a methodology that left room to discover unexpected themes.

## Conclusion

Clinicians broadly felt that the CF improved the quality of patient care as well as providers' professional satisfaction in their jobs. The CF mainly served to provide guidance and a pathway for continuous learning and problem-solving. Skills gained through application of the CF were also perceived to have improved community acceptance and utilization of services. Any future CF for the BPOC should therefore be considered important at both provider and systems levels, with the potential to both improve the consistency and quality of treatment as well as the resilience of providers in historically challenging roles. As is increasingly the case in medical and dental education, a CF should be integral to implementation of BPOC training, delivery and evaluation, and more translational research is required in this area. Further, the CF was perceived as closely intertwined with ongoing professional development courses, and structured continuous learning should be viewed as an essential part of implementing any CF for the BPOC effectively. These strategies will likely be most meaningful if recognized by government and other official licensing bodies, which can offer credentialing that has universal legitimacy in the healthcare field, and can provide validation of learning, increased stature, and job security for frontline BPOC providers. These processes may finally help the BPOC achieve its full potential in efforts to establish universal oral healthcare services where they are needed most.

## Data Availability Statement

Additional original data is presented in Supplementary Material. Further inquiries can be directed to the corresponding author.

## Ethics Statement

This study received ethical approval from the Nepal Health Research Council (Kathmandu, Nepal) and an exemption as non-human subjects research from the Institutional Review Board at the Johns Hopkins Bloomberg School of Public Health (Baltimore, MD). All respondents provided written informed consent to participate in this investigation.

## Author Contributions

BK and SA led this study, developing the research plan, obtaining ethical approval in Nepal, conducting all fieldwork, locating and collaborating with professional transcription/translation, leading analysis, and interpretation of data. LS contributed heavily to literature review, supported analysis, and took a lead role in drafting manuscript in English for team feedback. RM and DE contributed substantially to research design and methodology, early stages of analysis, and providing editorial and conceptual feedback as drafts of the manuscript emerged. All authors contributed to the article and approved the submitted version.

## Conflict of Interest

BK, SA, and LS declare a possible conflict of interest as members of Jevaia Foundation, which supported this study with internal program funds, partially employs or otherwise professionally supports study participants, and is the agency responsible for implementing the the Competency Framework that was evaluated in this investigation. The remaining authors declare that the research was conducted in the absence of any commercial or financial relationships that could be construed as a potential conflict of interest.

## Publisher's Note

All claims expressed in this article are solely those of the authors and do not necessarily represent those of their affiliated organizations, or those of the publisher, the editors and the reviewers. Any product that may be evaluated in this article, or claim that may be made by its manufacturer, is not guaranteed or endorsed by the publisher.
