# Supplementary material for: A Clinical Competency Framework for the Basic Package of Oral Care: Perceptions of Primary Oral Health Providers in Rural Nepal
Source: Front Public Health. 2022 Jul 14;10:914581. doi: 10.3389/fpubh.2022.914581 (PMC9330375; doi:10.3389/fpubh.2022.914581)
Supplement: Supplementary file 1 [file Data_Sheet_1.PDF]

Understanding the value of a competency framework for the Basic Package of Oral Care: A qualitative case study of primary oral health providers in rural Nepal

Bidhya Koirala, Shreedhar Acharya, Laura Spero, Rakhi Mittal & Daniel Erchick

Supplemental Materials

Section 1: BPOC Competency Framework & Annual Practicing Requirements……………………..….. p.1

Section 2: A Priori Codes used for first phase of analysis……………………..…………………….. p.1

Section 3: Discussion guides ………………………………………………………...……….p. 2

Section 4: Full presentation of representative quotes………………………………………………. p.4

1. COMPETENCY CHECKLISTS

Please access Competency Framework materials at
https://tinyurl.com/bpoccf

2. A PRIORI CODES USED FOR INITIAL ANALYSIS

**#**	**Code name**	**Topic areas / notes**
**1**	Objective of the research	Neutral
**2**	Previous experience	Neutral**.** To be used in participant description.
**3**	Roles/responsibilities	Neutral. Only describes what the interviewee does. We will use it to describe.
**4**	Guidance/Structure/Confidence	A sense of understanding what you're supposed to be doing<br>Comments about learning, learning process, learning mentality
**5**	Motivation/Job Satisfaction/Professionalism (internal to dental field)	Peer interaction, belief in self<br>Understanding or seeking a standard of care<br>Mutual learning and accountability with professional peers
**6**	Quality of Care / Specific skills	Sub-divisions: Health outcomes, Patient counseling, Safety, Treat, planning/Management, Referral. Also, specific skills learned/improved.
**7**	Professionalism (external to dental field) / Community Acceptance & Opinions	How others feel about their role, belief by others<br>Use of service (attendance) participation in treatment (accept interventions)
**8**	Professional development generally (not related to the CF) / Implementation of the framework / Supervision	The process of using the framework. Activities around the framework.
**9**	Challenges	Could be about the framework or not. Areas that the framework doesn't address, but might in the future.
**10**	Recommendations	Government advocacy for integration;<br>Legitimacy in dental field and government sphere;<br>Changes in implementation

3. DISCUSSION GUIDES

POHP Discussion Guide

1. **1. Warm-up**

   Let's start by having you tell me a little about yourself.

   1.1. 1Can you please share with me your role in this organization?

   1.2. What is your daily routine after you reach your various working areas?

2. **How Competency framework is used**

   I understand that in your role, you've been using a competency framework for about two years. What has been your experience with the competency framework?

   2.1. How were you trained on the competency framework?

   2.2. Do you use the competency framework in your day to day work, or not? If so, how?

   2.3. How did the competency framework change (older technicians) / affect (Hira) how you work with patients?

3. **Self-Confidence & Community Acceptance**

   3.1 Does the competency framework influence how you feel about your work? How?

   3.2 Do you think the competency framework influenced how other people feel about your role? How?

4. **Impact on clinical care**

   4.1. Did the competency framework improve the quality of care you provide? How?

   4.2. Would you like to share any examples of when:

      4.2.1. competency framework helped you with diagnosis

      4.2.2. helped you with treatment planning

      4.2.3. helped you with patient interaction, counseling, or comforting

      4.2.4. helped increase safety for you and/or the patient

      4.2.5. helped in some other way

5. **Impact on implementation of BPOC skills in the community**

   Now I would like to ask some technical questions related to the competency framework.

   5.1. Did the competency framework improve your clinical skills? How?

      4.1.1. Can you give a specific example of a procedure the competency framework helped you with?

   5.2. Did it help you to structure your workflow while seeing patients in the community? How?

      5.2.1. Can you give a specific example of how the competency framework has influenced your workflow?

   5.3. Did the competency framework impact your treatment planning, or affect how you decide what procedures to use?

      5.3.1. Can you give an example of a time you changed your treatment planning based on competencies you've learned?

6. **Impact on professionalism**

   6.1. Has the competency framework influenced:

      6.1.1. discussion/teamwork with your peers/dental professionals

      6.1.2. your expectations of yourself in your role

      6.1.3. your style of learning

7. **Challenges**

   7.1. Have you found any problems with the competency framework?

   7.2. What suggestions do you have for how the competency framework and annual renewal process could be improved?

8. **Recommendations for long term compliance**

8.1. In your view, what if any role should the competency framework have in oral health care delivery in Nepal?

8.1.1. What are your reasons for this recommendation?

9. **Is there anything else you'd like to share about your experience with the competency framework?**

10. **Do you have any questions?**

<u>Clinic Assistant Discussion Guide</u>

1. **Warm-up**

   Let's start by having you tell me a little about yourself.

   1.1. Can you please share with me your role in this organization?

   1.2. What is your daily routine after you reach your various working areas?

2. **General impressions of Competency framework**

   I understand that the technician you work with has been using a competency framework for about two years

   2.1. Since the competency framework was started, what changes did you notice in the working style of your technician?

   2.2. Did the competency framework affect your personal working style, or how you work with your technician? How?

   2.2.1. Can you give a specific example of a time when that happened?

   2.3. Do you think the competency framework influenced how other people feel about the oral health care service you provide in the community?

3. **Improved standard of care**

   3.1. Did the competency framework improve the quality of care you and your technician are providing? How?

   3.2. Would you like to share any examples/incidents when?

   3.2.1. competency framework helped your technician with diagnosis

   3.2.2. helped your technician with treatment planning

   3.2.3. helped you or your technician with patient interaction, counseling, comforting

   3.2.4. helped increase safety for you and/or the patient

   3.2.5. helped in some other way (for either you, your technician or your patient)

4. **How competency framework has improved BPOC delivery**

   4.1. Can you give an example of something you started doing differently after the competency framework was introduced? For example:

   4.1.1. Something related to clinical procedures?

   4.1.2. Something related to your workflow at community programs or schools?

   4.1.3. Something related to how you interact with your technician or with patients?

5. **Improved professionalism**

   5.1. What is your experience with the professional development training where competencies are discussed?

6. **Challenges**

   6.1. Have you found any problems with the competency framework?

   6.2. What suggestions do you have for how the competency framework and annual renewal process could be improved?

7. **Recommendations for long term compliance**

7.1. In your view, what if any role should the competency framework have in oral health care delivery in Nepal?

7.1.1. What are your reasons for this recommendation?

8. Is there anything else you'd like to share about your experience with the competency framework?

9. Do you have any questions?

4. FULL PRESENTATION OF REPRESENTATIVE QUOTES

1. <u>What is the Competency Framework to Me</u>

"We move our feet even on roads we have never taken."

Clarity of standards	● *What I feel about the competency framework is that we tend to move our feet even on roads that we have never taken. We might not arrive on time, but we will get there. What I feel about this framework is that while walking on a black, paved, straight road it might seem like we don't get very far. But if we walk kilometer-by-kilometer then we will reach the destination as planned without any wastage of time. (POHP)*<br>● *After the CF training, I started doing things step-by-step. (POHP)*<br>● *After attending the three weeks of training, it was much easier to work. I also felt comfortable working with the POHP as I knew his working procedure, which was easy to follow after the training. (CA)*
Learning Mindset	● *What I think is, whenever we work, we work in the same way that we are used to working. This competency training will show us how we can work by different ways or mediums or methods. This is very good for POHPs and assistants like us. It is very beneficial. (POHP)*<br>● *I used to do it in my own way but later after the training, I knew about the ways of asking the question and carrying out treatment accordingly. (POHP)*<br>● *I used to have a feeling that I may not be able to answer all the queries asked by the patients. This curiosity encouraged me to ask questions during the training. The instructor answered all my questions without hesitation. It has made a positive impact. (POHP)*

2. <u>Relationship to the Work</u>

"Words used to disappear from my mouth."

a. Confidence

"My hands used to tremble."

Increased confidence	● *I used to get scared even to speak. I used to be worried about work. I used to feel that I might make mistakes. After the training. I realized that I must talk with the patients and started asking about their purpose of visit. My communication skills have really improved. (CA)*<br>● *It was really difficult at the start as I had never done this work. One week of training was not enough. I used to hesitate if there was a male patient. I used to be scared, my hands used to tremble, and I couldn't even look at the patient's face as I used to be shy. Words used to disappear from my mouth. If it was another type of disease, I could have worked from afar. Dental service requires close contact…As I started participating in various training, I felt comfortable gradually to speak, to treat even the male patients." (POHP)*<br>● *If sometimes we are alone and something happens to the patient if we can work in those*

	situations then it's only because of Competency. (POHP)

b. Peer Accountability

"We discuss among friends."

Guidance-seeking for standard of care	• *After competency training, we have formed a group which contains people working in different places and when we have a problem…we discuss among friends and treat the patient next week. (POHP)*<br>• *In a situation when I didn't know something or was confused or not sure of something, it helped me to ask my seniors or friends. I could effectively talk with them. (POHP)*<br>• *After going to training we got to know everyone from different places. After that, we started speaking. (CA)*

c. Job satisfaction and motivation

"If I learn new things, it will motivate me."

Intellectual stimulation	• *If you receive training and utilize it for a long time it becomes an old technique. For example, if you start something in the morning it will be so fresh and energetic, slowly in the evening energy level goes down. Similarly, if I received training 12 years back and continue working with the same I won't learn new things and I might forget many things also. We need refresher training to update our knowledge and motivate us to work. (POHP)*<br>• *Rather than doing the same thing, if we learn new things it will motivate us. (POHP)*
Collegial support	• *We will have a feeling that 'I can do' when we see our friends doing things we find difficult. It motivates us to do such difficult work, which allows us to try and learn new things. (CA)*<br>• *We met friends from different places and shared our experiences. They knew the things which we didn't know, and we knew the things that they didn't know. (POHP)*
Competence related fulfillment	• *I used to be scared the POHP would notice faults in my work. Now after the training, I know where and how to keep things properly. I also know how to choose the right tool for the right treatment. I felt this made my work easier and comfortable. (CA)*<br>• *In the beginning, there used to be problems like leakage or shrinking or early falling out of ART fillings. But after receiving the second and third training, I found the filling that I had done a year earlier in more or less the same condition. This makes me feel that the training has really been great. (POHP)*

3. <u>Practical Improvements</u>

"There is a difference between knowing and doing things practically."

a. Clinical Skills

"The training has brought changes in our working nature and procedure."

Infection Control	• *We used to clean the clinic, instruments, chairs and other things that we use before the training, but we didn't do it regularly. After the training, we are doing all these activities regularly as a mandatory thing. (CA)*

	● *I used to go to bring the tools in the middle of treatment if the assistant was absent before the training. I learned that it is not a good idea to leave the patient once the hand is inserted in the mouth to pick up the tools. (POHP)*<br>● *We used to touch anywhere while wearing gloves and equipment would get contaminated.… After the training, we wear gloves and touch only the equipment. (CA)*
Direct treatment skills	● *It's not easy to do practically without proper training. There are things like how to dry up the teeth or apply cotton or how to extract the teeth, hold forceps, how to ensure sterilization. There is a difference in knowing and doing things practically. (POHP)*<br>● *In the past, I didn't know which side would have less saliva while putting in the cotton. After the training, I knew that. Likewise, I knew where to inject LA for an extraction, and that if it is given in the wrong place, it could cause pain. I also knew about holding Forcep during extraction and using the right Forcep for the right teeth. (POHP)*<br>● *We realized only after the training that the patients had pain after filling teeth if we failed to clean the inside of the tooth properly. (POHP)*<br>● *The filling used to drop out soon after the ART due to a mixing problem. It was a major problem as it frequently happened….You taught us how to hold it from the side, which made our work way better. (POHP)*<br>● *The POHP used to come and point out the needed instruments as I was unable to recognize them before the training. But now I could take the items needed for her when she simply asks while she is busy with the patients. I could also identify what treatment is done to the patient. (CA)*
Differential Diagnosis & Referral	● *There has been a huge improvement in the quality….We didn't know when to do the filling, which has improved. As in, in this case, this should be done. We do things that we can and refer cases we can't deal with. (POHP)*<br>● *Before the training, the POHP I work with used to try to treat all sorts of cases though he didn't know how to. He was not in favor of referring to the hospital and tried to do it himself. After the training, a lot of changes have been seen. He refers many patients to the hospital and encourages them to go. (CA)*<br>● *Now, we know what treatment is required for what case and when to carry out or not to carry out the certain treatment. (POHP)*<br>● *It helped to provide the treatment according to their history. It even helped us to determine what treatment should be required for which teeth, which eased our work. (??)*<br>● *I used to use SDF only if the cavity was big or GIC was not possible. Later on, I realized that full mouth SDF was better. (POHP)*<br>● *After the training we were able to distinguish various conditions to apply SDF or Fluoride or ART. This really made our work easy. (POHP)*
Approach to care	● *We used to go there with a fixed responsibility just to extract the teeth, fill the teeth. After this training when we started to work for certification I started to see the patient from a different perspective. Patients are God. (POHP)*<br>● *After going into the competency framework there has been a perspective change of us towards patients. Like before when patients came for treatment we used to think that they might have just come due to tooth pain and we can do more later, too. But now we think that if we treat an issue today, the patient won't come back with the same problem. We hope that it will make changes in the daily lifestyle of the patient. (POHP)*

b. Patient Communication

"It has brought a lot of changes in my working style."

Effective patient communication	● *It has brought a lot of changes in my working style….When a child sees a man with a white coat, the child gets scared. We have to build a capacity to deal with such cases. (POHP)*<br>● *I knew how to communicate with a patient who is suffering from a toothache. I knew how to convince patients who just want to extract their teeth no matter what the case is. I became mature in counseling. (POHP??)*<br>● *The change is quite evident…The POHP properly asks what needs to be done. We also explain the things that we know to them. (CA)*<br>● *There have been changes starting when patients enter. We wipe down the chair and make the person comfortable…We clear all their doubts and ask them how they are feeling. We make them really confident and drive their fear away. (CA)*<br>● *The POHP also kindly talks with the patients. He asks everything in detail. He asks them how they are feeling? How long have they been facing the problem? He behaves very well to the patients. (CA)*<br>● *When we received the counseling training, we learned the counseling skill and we started our treatment after proper counseling and the patients were ready and relaxed. (POHP)*

c. Workplace Relationships

"There is no debate about the work."

Teamwork	● *I used to think that I should do my work alone. I used to think that we all have separate work, and we should do it separately. But after the training, we started doing it collectively even while visiting the community. It made our work easier and better. Likewise, there was no debate about the work. There was cooperation. (POHP)*<br>● *Before we needed to show and teach which equipment needed to be brought to us. But now if I say to bring ART equipment, the assistant recognizes it and brings that equipment. (POHP)*<br>● *After attending the three weeks of training, it was much easier to work. I also felt comfortable working with the POHP as I knew his working procedure, which was easy to follow. (CA)*<br>● *Now, I can tell what is happening, how things are going. I can talk to the POHP face-to-face. (CA)*

<u>4. Community Impact</u>

"Now they come searching for us."

Community Acceptance	● *Around 11-12 years back when we started to work people did not have full confidence….They might have given us an opportunity only because they didn't have an option. But now they come searching for us to have the service. There is a perspective change of society while working. (POHP)*

	• *The last time we did a community program, people said afterwards that in previous programs they'd faced many difficulties, but now we'd done the program in a much more easy manner and everyone got checked easily. This is something I felt happy about. Before this we had these difficulties, but the competency has improved our management. (POHP)*
Crowd Management	• *After the training, I learned not to get panicked while working in the community in front of a large number of people. (POHP)*<br>• *I knew after the training that it was not possible to do all the work right in the field…. which made my work more comfortable and effective. The patients also understood it and it was advantageous for both. (POHP)*<br>• *In the past, the patients used to enter all at once making a crowd and they used to complain. Now, we keep them all out in an organized way. They talk about these changes with each other. (CA)*
Increased Participation	• *Initially we used to have very limited patients but now we receive all patients from this village and even many people come to our place referred by other places. (CA)*<br>• *The training made me comfortable that if the counseling is provided in this way, the patients are convinced to carry out the treatment. (POHP)*

Challenges

Operational challenges	• *It is difficult for us to give treatment by complying with the competency framework. Sometimes patients come in a hurry, saying "someone is babysitting my child," or breastfeeding a toddler and saying "I have to go home in 10 minutes." So should we take their history or treat them in 10 minutes? We get confused. (POHP)*<br>• *People often think "why should we go there if they won't even extract the mere teeth?" (POHP)*<br>• *Sometimes patients with chronic problems hide their disease, thinking that they won't get dental services if their pre-existing health issues are revealed. This makes the work difficult as it could cause bleeding or take a long time or even lead to a risky situation. (POHP?)*<br>• *No matter how good your work is, if the patient doesn't follow instructions, it won't work and then we must bear the blame. (POHP?)*
Credentialing challenges	• *People here think that we have not done dental studies, rather we are just a common POHP. That is possibly the reason, I think, behind their doubt. (POHP)*

Recommendations

" This organization has taught us many things, but hasn't given us any rights." (POHP)

Certificate validity	• *This certificate does not have validation with the Nepal government. I know this organization is doing good work and the public benefits directly and the work is very effective, but these procedures are not legitimized. The competency certificate you are*

	talking about now by Jevaia is not valid outside this organization. (POHP)<br>● *Even with a competency certificate, the work is the same and there is no upgrade in the post. So there is no difference between taking and not taking the competency certification. (POHP)*<br>● *If the government allocated a sanctioned position, we would feel that this [Competency] certificate is valuable. We have achieved the competency framework certificate but we don't know where it works or how to make use of it. (POHP)*
Job security	● *We don't see announcements like, "Seeking a skilled POHP at the local level who has a Competency Certification and BPOC training." For that we need a [government-] sanctioned position. (POHP)*<br>● *If an official post is added and there is a sanctioned position for the POHP, only then will the competency have a benefit. (POHP)*<br>● *The experience I have and this certificate are not something you just store in a cupboard. If you could clarify where we can use the certificate then we would be grateful. (POHP)*
